# Perinatal health care access, childbirth concerns, and birthing decision-making among pregnant people in California during COVID-19

**DOI:** 10.1186/s12884-021-03942-y

**Published:** 2021-07-02

**Authors:** Mackenzie D. M. Whipps, Jennifer E. Phipps, Leigh Ann Simmons

**Affiliations:** Department of Human Ecology, Perinatal Origins of Disparities Center, University of California, Davis, California USA

**Keywords:** COVID-19, SARS-CoV-2, Pregnancy, Perinatal, Healthcare access, Childbirth, Decision-making, California, Racial/ethnic minoritization, Financial insecurity

## Abstract

**Background:**

During public health emergencies, including the COVID-19 pandemic, access to adequate healthcare is crucial for providing for the health and wellbeing of families. Pregnant and postpartum people are a particularly vulnerable subgroup to consider when studying healthcare access. Not only are perinatal people likely at higher risk for illness, mortality, and morbidity from COVID-19 infection, they are also at higher risk for negative outcomes due to delayed or inadequate access to routine care.

**Methods:**

We surveyed 820 pregnant people in California over two waves of the COVID-19 pandemic: (1) a ‘non-surge’ wave (June 2020, *n* = 433), and (2) during a ‘surge’ in cases (December 2020, *n* = 387) to describe current access to perinatal healthcare, as well as concerns and decision-making regarding childbirth, over time. We also examined whether existing structural vulnerabilities – including acute financial insecurity and racial/ethnic minoritization – are associated with access, concerns, and decision-making over these two waves.

**Results:**

Pregnant Californians generally enjoyed more access to, and fewer concerns about, perinatal healthcare during the winter of 2020–2021, despite surging COVID-19 cases and hospitalizations, as compared to those surveyed during the COVID-19 ‘lull’ in the summer of 2020. However, across ‘surge’ and ‘non-surge’ pandemic circumstances, marginalized pregnant people continued to fare worse – especially those facing acute financial difficulty, and racially minoritized individuals identifying as Black or Indigenous.

**Conclusions:**

It is important for clinicians, researchers, and policymakers to understand whether and how shifting community transmission and infection rates may impact access to perinatal healthcare. Targeting minoritized and financially insecure communities for increased upstream perinatal healthcare supports are promising avenues to blunt the negative impacts of the COVID-19 pandemic on pregnant people in California.

**Supplementary Information:**

The online version contains supplementary material available at 10.1186/s12884-021-03942-y.

## Introduction and background

Healthcare access is a crucial barometer of population wellbeing worldwide. The ability to access care when one is ill, injured, or otherwise in need of medical support is a necessary condition for a community to survive and thrive in the twenty-first century [1]. During public health emergencies, whether a natural disaster, a severe economic downturn, or a pandemic, access to adequate healthcare remains an important metric to measure, and ultimately, an important lever for intervention [2].

The World Health Organization (WHO) officially declared the outbreak of COVID-19, the illness caused by the novel respiratory virus SARS-CoV-2, to be a pandemic on March 11th, 2020 [3]. Since that time, COVID-19 infection rates have increased exponentially across the world [4]. As of this writing, the nation with the highest infection rate by far is the United States: on June 1st of 2020, the United States had confirmed 1.8 million cases and 108,500 fatalities, and 6 months later, on December 1st, that number had grown to 13.8 million confirmed cases with 270,800 fatalities [5]. Healthcare systems across the country have been stretched to their limits. Personal protective equipment was scarce. Providers were working longer hours and more consecutive shifts while managing more complicated and critical cases. Patients were being doubled up in single rooms, boarded in makeshift rooms and hallways, or in the worst cases, being turned away from hospitals due to the lack of space, personnel, and equipment resources.

The locations of viral epicenters within the United States have shifted over time in response to a number of factors, including state and local public health policy initiatives to curb the transmission of COVID-19 in community hotspots. While New York City and other dense, urban areas were initial epicenters, infections have gradually spread to more remote areas. Rural and suburban areas are now over-represented in terms of both overall cases and fatalities [5, 6].

The state of California, in particular, is unique in this respect. With over 40 million residents, California was the first US state to impose strict ‘lockdown’ measures to curb COVID-19 outbreaks and prevent their spread to other areas [7]. On March 16th, only 5 days after the WHO declared COVID-19 a pandemic, the governor of California issued a shelter-in-place order for 6 counties; that order was extended statewide on March 19th [8]. These early measures were largely considered successful. Early adoption seems to have been more effective in terms of reductions in cases as compared to delayed action [9], and simulations have estimated that nearly 1500 deaths were averted in just the first 4 weeks of enactment, when case rates were still very low throughout the state [10]. By early May, many cities and counties in California began relaxing restrictions, with county-level infection rates holding steady or rising slowly throughout the summer and autumn of 2020 [5]. However, on December 3rd, statewide region-by-region shelter-in-place orders were reinstated as the state of California passed 1.25 million COVID-19 cases [8]. Hospitalizations soon reached an all-time high in the state, and capacity in intensive care units fell to dangerously low levels [11]. These ebbs and flows of case rates and associated hospitalizations over time – so-called ‘surges’ – represent specific historical and geographic contexts within which healthcare access in emergencies can be studied in-depth.

Pregnant people are a particularly vulnerable subgroup to consider when studying healthcare access in emergencies. Not only are pregnant people likely at higher risk for illness, mortality, and morbidity from COVID-19 infection [12, 13], they are also at higher risk for negative outcomes due to delayed or inadequate access to routine care. Timely and appropriate perinatal care from a qualified health professional is one of the primary determinants of a healthy pregnancy, a healthy birth, and later health outcomes for birthing people and their children [14, 15]. However, studies conducted during previous viral outbreaks have shown that barriers to healthcare utilization – especially reproductive and maternal/child healthcare – increase dramatically during public health emergencies [16]. During times of health crisis, such as a local ‘surge’ of infection during a pandemic, it is crucial that health practitioners and policy makers understand whether the healthcare needs of pregnant people are being met, whether other factors related to inequitable access to healthcare are being compounded by the acute crisis, and how best to support this vulnerable population.

Research is only just beginning to explore the indirect effects of the 2020–2021 COVID-19 pandemic on perinatal healthcare access and quality. Researchers in Europe and the United States have found increased concerns about perinatal health among pregnant people, including increased feelings of fear and worry and higher rates of pregnancy-related anxiety [17, 18]. Qualitative work with pregnant women in Turkey found that women reported skipping planned prenatal appointments and putting their prenatal care ‘on hold’ while the pandemic raged around them [19]. Some women also reported shifting plans for the birth of their child in response to increasing rates of COVID-19 in their local hospital [19].

In the United States, researchers who have surveyed healthcare providers have similarly found that pregnant people are expressing intense fear of contracting COVID-19 in a hospital setting [20]. Providers report reducing the number of prenatal visits, shifting to telemedicine-based prenatal care, and asking patients to take over responsibility for tracking vital health markers like blood pressure [20]. Like in Turkey, US providers and pregnant people also report a substantial increase in the number of pregnant people exploring childbirth options that limit their exposure to hard-hit local hospitals, including midwife-attended homebirths, birth-center births, and unattended ‘freebirths’ [17, 20]. Many healthcare providers postulate that this shift reflects a fear of the hospital and associated risk for contracting COVID-19 rather than a real desire for an out-of-hospital birth [20, 21]. Though out-of-hospital birth can be as safe as in-hospital births (and perhaps even safer than in-hospital births for certain low-risk individuals [22, 23]), it is generally recognized by home-birth and hospital-birth providers alike that planning an out-of-hospital birth out of fear or panic caused by rising rates of infection, or switching to a homebirth late in pregnancy, is not ideal [20, 21].

Pandemic-related worry and lower access to perinatal healthcare has been shown to be especially pronounced among minoritized and/or marginalized individuals [24]. These same populations also generally face increased barriers to accessing adequate perinatal care in high-income countries, even before the added obstacles of a public health emergency [25]. Understanding whether and how local infection rates and existing marginalization may interact to produce these worries, perceptions, and decisions regarding perinatal healthcare, especially in a locality with some of the most extreme COVID-19 infection rates in the world, is an important next step.

### Current study

Given the importance of perinatal healthcare access to family and community health, this study sought to describe the context of adversity facing pregnant people over 2 distinct timepoints during the 2020–2021 COVID-19 pandemic in California: a summer ‘non-surge’ wave and a winter ‘surge’ wave. We also sought to explore whether perinatal healthcare access, concerns, and decisions were different for subgroups with further vulnerabilities, including minoritized people and people facing acute financial difficulty. We undertook three specific aims toward these ends:

*Aim 1*. Describe overall rates and changes in rates of self-reported access to prenatal healthcare in California over 2 time points during the 2020–2021 COVID-19 pandemic.

*Aim 2*. Describe whether and how concerns and decision-making regarding childbirth have changed over 2 time points during the 2020–2021 COVID-19 pandemic.

*Aim 3*. Explore whether these adverse perinatal healthcare experiences differ by racial/ethnic minoritization and by individual levels of financial strain.

## Methods

### Participants

Our team surveyed pregnant Californians cross-sectionally using two web-based surveys. Wave 1 data were collected from June 6th to July 29th of 2020, while Wave 2 data were collected from December 24th 2020 to January 27th 2021. The recruitment materials and surveys were available in both English and Spanish, which are the top two most spoken languages in the state. Participants took approximately 20–30 min to complete the survey and were offered a gift card for their participation. We solicited participants using targeted social media campaigns (e.g., on Facebook, Instagram, Twitter) describing the study in English and Spanish and featuring diverse images of pregnant people and couples. Participants were considered eligible if they resided in California, were between ages 18 and 45 years, and were currently pregnant. Informed consent was obtained via electronic signature. Ethics approval was provided prior to the start of the study by University of California, Davis’ Institutional Review Board. All methods, including informed consent procedures, were carried out in accordance with the Declaration of Helsinki – Ethical Principles for Medical Research Involving Human Subjects.

#### Missing data

Several strategies to ensure high data quality were employed for this web-based survey. Participants who did not reach the end of the survey instrument, completed the survey in less than 10 min, or attempted to take the survey multiple times had their data removed for analysis. Participant age was asked twice, once at the beginning of the survey and once at the end; any participant without matching responses for these two items was removed from analyses. In all, 216 participants were not included in the analytic sample as a result of these quality checks (155 out of 588 in Wave 1 and 62 out of 454 in Wave 2). Sporadic missingness was handled using list-wise deletion.

### Measures

All survey items within the questionnaire can be found in the supplementary file.

#### Prenatal healthcare access

Four items assessed participants’ current access to prenatal healthcare experiences. Participants were asked: “Has your provider started doing remote visits, such as using video or telephone?” (responses were dichotomous, yes/no) and “Has your provider reduced the number of visits?” (responses were dichotomous, yes/no). For both items, participants also had the option to select “I don’t know” and “I prefer not to answer”.

Participants also were asked to respond to the following prompt to assess how often and for what reasons they accessed healthcare during their prenatal period: “Please rate the following statements in terms of how you have been doing for the last 7 days.” These items were: “I seek care for health problems not related to my pregnancy” and “I schedule an extra visit with my prenatal provider if I am concerned about my pregnancy”. Response options were a Likert-type, behaviorally anchored scale which ranged from 0 to 4: 0 - *Never, 0 days*; 1 - *Rarely, 1–2 days*; 2 - *Sometimes, 3–4 days*, 3 - *Often, 5–6 days*, and 4 - *Always, 7 days*. Participants also had the option to select “Does not apply” and “I prefer not to answer”.

#### Concerns about childbirth healthcare access

Similar to previous items, participants reported how often they were worried or concerned about the following issues related to their childbirth: “I worry that I will not have my birth support person with me in the delivery room”; “I worry that my provider or health care team will not be available during my delivery”; and “I worry that my healthcare team will not have the equipment and resources they need to support my delivery”. As above, response options ranged from 0 (*Never*) to 4 (*Always*) and were treated as continuous in analyses. A measure of Cumulative Concerns about Childbirth Healthcare Access was also created by summing responses to the three individual concerns; the range of the cumulative measure was 0–12.

#### Childbirth decision-making

One item asked participants to report how often they considered having an out-of-hospital birth for their current pregnancy (“I am thinking about not having my baby in a hospital”), again with response options ranging from 0 (*Never*) to 4 (*Always*). Participants also were asked whether they had planned to give birth outside of the hospital (yes, no, prefer not to answer) and if so, where (home, birth center, other). Those participants who indicated that they were planning an out-of-hospital birth were then prompted to respond to the following question: “Did COVID-19 change your plans for where to deliver your baby?” (yes, no, prefer not to answer).

#### Racial/ethnic Minoritization

Racial/ethnic minoritization, a structural variable that seeks to index exposure to racism and discrimination based on being a visible minority in a white supremacist culture, was conceptualized in a number of ways. At its most broad, we operationalized minoritization as holding a non-white and non-Hispanic identity. Therefore, white participants are compared against those who identify as people of color (POC), and non-Hispanic participants are compared to Hispanic participants. Exploratory analyses also broke down the larger category of ‘racially minoritized’ into specific racial categories: 1 - *Black / African-American only*, 2 - *American Indian / Indigenous / First Nations only*, 3 - *Asian / Asian-American / Native Hawaiian / Pacific Islander only*, 5 - *Some other race only*, and 6 - *Multiracial*. There was an inadequate number of Native Hawaiian and Pacific Islanders to analyze separately as their own group, thus they were grouped with Asians and Asian Americans.

#### Financial insecurity

Financial insecurity was operationalized using a single survey item, which asked participants “During the past 2 months, how much difficulty have you had paying your bills?”. Responses ranged from 1 to 5, with the following anchors: 1 – *No difficulty at all*; 2 – *A little difficulty*; 3 – *Some difficulty*; 4 – *Quite a bit of difficulty*; and 5 – *A great deal of difficulty*. Participants also had the option to select “I prefer not to answer”. Responses are treated as continuous in analyses.

### Data analysis

#### Access, concerns, and decision-making across waves

First, we described access, concerns, and decision-making across data collection waves. We conducted *χ*^2^ tests and univariate regressions to test for differences by wave. We hypothesized that measures of access would decrease, and measured concern would increase, during times of ‘surge’ (the winter wave). We did not have directional hypotheses with regard to childbirth decision-making (i.e., planning or considering an out-of-hospital birth).

#### Differences by racial/ethnic minoritization and financial insecurity

First, we examined correlations between the constructs of interest for each wave of data collection. Next, we utilized multivariate regression-based analyses to predict our outcomes of interest (access, concerns, and decision-making) from our predictors of interest: racial/ethnic minoritization and financial insecurity on a combined sample that included both waves of data, controlling for participant age, primiparity, urbanicity, and essential worker status. Sensitivity analyses were conducted to ascertain whether these relationships differed by data wave. Finally, we descriptively probed the findings on childbirth concerns plotting the cumulative measure of childbirth concerns across wave, ethnic minoritization, level of financial insecurity, and racialized identities. Given the small absolute numbers of participants within each of the specific categories – especially within each racial and financial insecurity subgroup – these further analyses are considered exploratory and hypothesis testing was not conducted.

## Results

The analytic samples of pregnant Californians are comprised of 433 participants for wave 1 (summer 2020) and 387 participants for wave 2 (winter 2020–2021). See Table 1 for demographic characteristics of analytic samples in both data waves. Samples are similar across waves, with some small differences: wave 2 participants are on average ~ 1 year older, earlier in their pregnancies, more likely to be essential workers, and live in more urban contexts than wave 1 participants. Race and ethnicity demographics for this sample are very similar to the demographic makeup of California at large [26].

**Table 1:** Demographic characteristics across data collection wave

Characteristic	June–July 2020 *n* = 433 N (%)	December 2020–January 2021 *n* = 387 N (%)
Maternal age		
18–24	53 (12.2)	27 (7.0)
25–34	255 (58.9)	224 (57.9)
35+	125 (28.9)	136 (35.1)
Participant is essential worker		
Yes	103 (23.8)	149 (38.5)
No	112 (25.9)	84 (21.7)
Not currently employed / no answer	218 (50.3)	154 (39.8)
Ethnicity		
Hispanic	149 (34.4)	151 (39.0)
Not Hispanic	280 (64.7)	236 (61.0)
Race		
White	233 (53.8)	199 (51.4)
Black / African American	20 (4.6)	18 (4.7)
Indigenous / First Nations	4 (.9)	2 (.5)
Asian / Pacific Island / Native Hawaiian	36 (8.3)	37 (9.6)
Other race	37 (8.6)	42 (10.9)
Multiracial	82 (18.9)	64 (16.5)
Urbanicity		
Rural	22 (5.1)	14 (3.7)
Semi-rural	51 (11.8)	33 (8.6)
Suburban	191 (44.1)	162 (42.3)
Urban	99 (22.9)	85 (22.2)
Major metropolitan	63 (14.6)	89 (23.2)
Trimester of pregnancy		
1st (0–13 weeks+ 6 days)	96 (22.2)	121 (31.3)
2nd (14–27 weeks+ 6 day)	188 (43.4)	172 (44.4)
3rd (28–42 weeks)	142 (32.8)	94 (24.2)
No answer / unsure	7 (1.6)	0 (.0)
Parity		
Primipara	206 (47.6)	183 (47.4)
Multipara	227 (52.4)	203 (52.6)

Every participant did not respond to every item; therefore Ns do not necessarily sum to total analytic sample size

### Access, concerns, and decision-making across waves

**Fig. 1:**
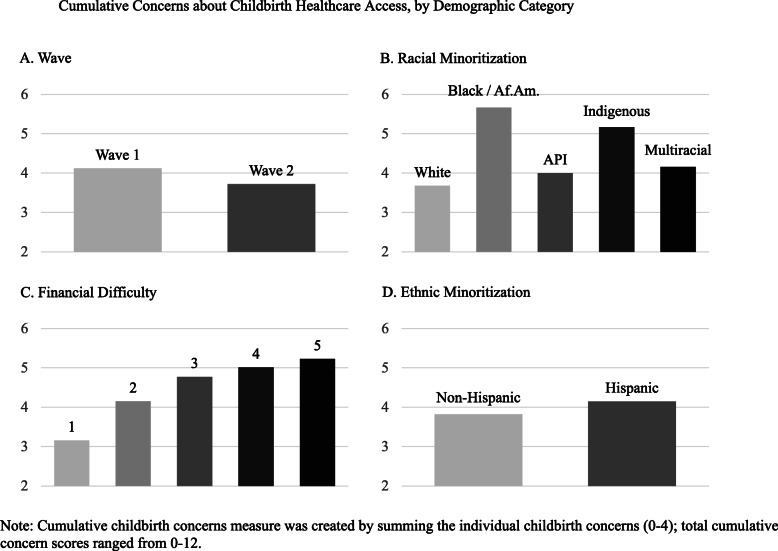
Cumulative Concerns about Childbirth Healthcare Access, by Demographic Category

Table 2 shows the results for access, concerns, and decision-making across waves. We found that prenatal healthcare access was overall better during wave 2 (the winter ‘surge’) than during wave 1. Fewer participants reported that their provider had reduced the number of prenatal appointments, though more participants were unsure whether their providers had done so. Pregnant participants also sought care for health issues outside of pregnancy more often, and there was a trend toward participants scheduling extra prenatal appointments if concerned as well. We found only one difference in the frequency of childbirth concerns across waves: participants in the first wave reported more frequent worrying about not having access to a support person during their birth than those surveyed during the second wave (wave 1 mean = 2.2, SD = 1.3; wave 2 mean = 1.8, SD = 1.4; *β* = − 0.39, *p* < 0.001). Cumulative concerns about childbirth, however, were similar across data collection waves (Fig. 1A).

**Table 2:** Snapshot of healthcare access, childbirth concerns, and childbirth decision-making across data collection wave

	June–July 2020 *n =* 433 N (%)	December 2020–January 2021 *n =* 387 N (%)	*p*-value	Sig.
**Prenatal healthcare access**				
Provider has begun remote visits			0.10	
Yes	190 (43.9)	148 (38.2)		
No	191 (44.1)	175 (45.2)		
Unsure / no answer	52 (12.0)	64 (16.5)		
Provider has reduced number of prenatal visits			< 0.001	***
Yes	147 (34.0)	90 (23.3)		
No	209 (48.3)	183 (47.3)		
Unsure / no answer	77 (17.8)	114 (29.5)		
I seek care for health issues outside pregnancy			0.02	*
Always	46 (10.6)	43 (11.1)		
Sometimes / Often	108 (24.9)	122 (31.5)		
Rarely / Never	240 (55.4)	186 (48.1)		
Does not apply / I don’t know	39 (9.0)	36 (9.3)		
I schedule extra prenatal visits if concerned			0.06	t
Always	60 (13.9)	57 (14.7)		
Sometimes / Often	75 (17.3)	85 (22.0)		
Rarely / Never	231 (53.4)	183 (47.3)		
Does not apply / I don’t know	67 (15.5)	62 (16.0)		
**Concerns about childbirth healthcare access**				
I worry about missing support person during birth			< 0.001	***
Always	89 (20.6)	65 (16.8)		
Sometimes / Often	208 (48.0)	143 (37.0)		
Rarely / Never	130 (30.0)	169 (43.7)		
Does not apply / I don’t know	6 (1.4)	10 (2.6)		
I worry about provider being unavailable during birth			0.82	
Always	19 (4.4)	19 (4.9)		
Sometimes / Often	121 (27.9)	99 (25.6)		
Rarely / Never	281 (64.9)	252 (65.1)		
Does not apply / I don’t know	12 (2.8)	17 (4.4)		
I worry provider won’t have resources during birth			0.82	
Always	16 (3.7)	18 (4.7)		
Sometimes / Often	90 (20.8)	80 (20.7)		
Rarely / Never	320 (73.9)	276 (71.3)		
Does not apply / I don’t know	7 (1.6)	13 (3.4)		
**Childbirth decision-making**				
I am thinking about not having my baby in a hospital			0.06	t
Always	21 (4.9)	15 (3.9)		
Sometimes / Often	52 (12.0)	33 (8.5)		
Rarely / Never	338 (78.1)	319 (82.4)		
Does not apply / I don’t know	22 (5.1)	20 (5.2)		
Planned location of birth			0.35	
Hospital	402 (92.8)	368 (95.6)		
Birth center	19 (4.4)	12 (3.1)		
Homebirth	6 (1.4)	3 (.8)		
Unsure / no answer	6 (1.4)	2 (.5)		
COVID-19 changed my plans for where to give birth ^a^			1.00	
Changed plans to birth center	2 (8.0)	2 (13.3)		
Changed plan to a homebirth	3 (12.0)	1 (6.7)		
COVID did not change planned place of birth	20 (80.0)	12 (80.0)		

^a^Only asked of those planning an out-of-hospital birth

^b^*p-*value of univariate OLS regression for continuous variales, Chi-squared test for dichotomous variables

^t^*p <* 0.10, *p < 0.05, **p < 0.01, ***p < 0.001

There was a trend toward considering having an out-of-hospital birth more often during the first wave, as compared to the second wave, for these participants as well (wave 1 mean = 0.63, SD = 1.1; wave 2 mean = 0.49, SD = 1.0; *β* = − 0.14, *p* = 0.061). However, rates of *planned* out-of-hospital birth remained statistically the same regardless of data collection wave (less than 10% at each wave), as did reporting that COVID-19 had changed their plans for where to birth (20% of those planning an out-of-hospital birth in both waves).

### Differences by racial/ethnic minoritization and financial insecurity

Cross-sectional correlations by data collection wave showed that racial/ethnic minoritization and financial insecurity were significantly related to several perinatal healthcare access outcomes of interest (see Appendix Table 1 and 2). This was especially true for concerns regarding accessing healthcare at the time of childbirth: those who are racially or ethnically minoritized, and those who experienced acute financial insecurity, were more often concerned about having a support person available, having their provider available, and having access to adequate resources during childbirth.

Using multivariate ordinary least-squares (OLS) and logistic regressions (Table 3), we found that racial minoritization (broadly defined) did not have large or significant associations with perinatal healthcare access, concerns about access during childbirth, or considering / planning an out-of-hospital birth, with one exception: after controlling for financial insecurity, ethnic minoritization, age, urbanicity, parity, and essential worker status, racially minoritized participants (as compared to white participants) were more likely to worry about having access to equipment or resources during birth. However, when disaggregated by specific racial identity, a more complex picture emerged. Using a cumulative measure of all three childbirth concerns, we found that Black and Indigenous pregnant people, specifically, were more often concerned about accessing appropriate care when compared to those identifying as white, Asian or Pacific Islander, or multiracial (see Fig. 1B).

**Table 3:** Independent models predicting healthcare access, childbirth concerns, and childbirth decisionmaking (*n* = 820)

**Predicting perinatal healthcare access**				
	β (Std. Err.)	*p-*value	Sig.	Cohen’s D
I schedule extra prenatal visits if concerned				
Minoritization (POC)	0.06 (.12)	0.617		0.05
Hispanic ethnicity	0.16 (.13)	0.233		0.11
Difficulty paying bills	−0.02 (.05)	0.740		−0.01
I seek care for health issues outside pregnancy				
Minoritization (POC)	−0.11 (.13)	0.415		−0.07
Hispanic ethnicity	−0.32 (.14)	0.021	*	−0.22
Difficulty paying bills	0.10 (.06)	0.067	t	0.07
	AOR (Std. Err.)	*p-*value	Sig.	
Provider has begun remote visits				
Minoritization (POC)	0.83 (.15)	0.311		
Hispanic ethnicity	1.07 (.21)	0.710		
Difficulty paying bills	1.06 (.08)	0.451		
Provider has reduced number of prenatal visits				
Minoritization (POC)	0.75 (.15)	0.158		
Hispanic ethnicity	0.82 (.17)	0.342		
Difficulty paying bills	1.29 (.1)	< 0.001	***	
**Predicting concerns about childbirth healthcare access**				
	β (Std. Err.)	*p-*value	Sig.	Cohen’s D
I worry about missing support person during birth				
Minoritization (POC)	−0.02 (.11)	0.851		−0.02
Hispanic ethnicity	0.07 (.12)	0.567		0.05
Difficulty paying bills	0.18 (.05)	< 0.001	***	0.13
I worry about provider being unavailable during birth				
Minoritization (POC)	−0.13 (.1)	0.185		−0.11
Hispanic ethnicity	0.01 (.1)	0.899		0.01
Difficulty paying bills	0.17 (.04)	< 0.001	***	0.14
I worry provider won’t have resources during birth				
Minoritization (POC)	−0.24 (.08)	0.006	**	−0.22
Hispanic ethnicity	−0.20 (.09)	0.028	*	−0.18
Difficulty paying bills	0.19 (.04)	< 0.001	**	0.17
**Predicting childbirth decision-making**				
	β (Std. Err.)	*p-*value	Sig.	Cohen’s D
I am thinking about not having my baby in a hospital				
Minoritization (POC)	−0.01 (.09)	0.922		−0.01
Hispanic ethnicity	−0.13 (.1)	0.163		−0.13
Difficulty paying bills	0.14 (.04)	< 0.001	***	0.13
	AOR (Std. Err.)	*p-*value	Sig.	
Planned out-of-hospital birth				
Minoritization (POC)	1.14 (.43)	0.734		
Hispanic ethnicity	0.43 (.21)	0.082	t	
Difficulty paying bills	0.97 (.16)	0.850		

In addition to constructs of interest, all models control for maternal age, essential worker status, urbanicity, and parity

^t^*p* < 0.10, *p < 0.05, **p < 0.01, ***p < 0.001

Ethnic minoritization (specifically, Hispanic identity) was significantly predictive of lower likelihood of seeking perinatal healthcare outside of pregnancy concerns and was associated with less worry about accessing equipment or resources during childbirth as compared to non-Hispanic participants. All other associations were not significant at the *p* < 0.05 threshold. Further, differences by ethnic minoritization were not apparent in cumulative childbirth concerns (see Fig. 1D).

Finally, acute financial insecurity was significantly related to a number of indicators of lower perinatal healthcare access, after controlling for relevant demographic characteristics and racial/ethnic minoritization. Each unit increase in financial insecurity was predictive of a 29% higher likelihood of reporting a reduced number of prenatal appointments. Increasing acute financial insecurity was significantly related to all three concerns about accessing healthcare during childbirth (Table 3), as well as the cumulative measure of childbirth concerns (Fig. 1C). Pregnant people with more financial insecurity also reported considering an out-of-hospital birth more often than those with fewer financial vulnerabilities. Each unit increase in financial insecurity was associated with a relatively small decrease in access, however (Cohen’s D all between 0.10 and 0.20 standard deviations).

To explore whether these associations were similar across data collection waves, we also split the sample into wave 1 (*n* = 433) and wave 2 (*n* = 387) to conduct these multivariate regression-based analyses. These sensitivity analyses showed that over both waves, the magnitude and direction of the findings did not substantially differ. However, because of the significantly reduced sample sizes, significance levels of findings were diminished.

## Discussion

The current study examined whether and how perinatal healthcare access for pregnant Californians has changed throughout the course of the COVID-19 pandemic. Specifically, we examined differences in access, concerns, and decision-making between ‘non-surge’ (summer) and ‘surge’ (winter) waves of data collection. Contrary to our hypothesis, we found that prenatal healthcare access remained the same or improved from an earlier, ‘non-surge’ context to a later, ‘surge’ context. To place these data in context for California, in the previous year (2019) 78.7% of live births were to pregnant people who received adequate prenatal care, 12.1% were to pregnant people with intermediate care, and 9.1% were to pregnant people who received inadequate care [27]. During the winter surge, pregnant people also were less concerned about lacking a support person during childbirth, and thought about having an out-of-hospital birth less often, despite rapidly rising COVID-19 cases and hospitalizations in California. These unexpected results could reflect a number of contributing factors. In the first wave, we were still learning about the virus, including routes of viral transmission, and recommendations for the best and necessary mitigation strategies were mixed. By the second wave, science was clear on aerosol transmission as the primary vector. Accordingly, healthcare providers had better prevention strategies in place for both outpatient and inpatient clinical care. More effective treatments for COVID-19 infection were also available, perhaps reducing the initial fear associated with the virus. Another contributing factor to wave 2 findings may be that 9 months into the pandemic, respondents were experiencing ‘pandemic fatigue’, or the general demotivation to follow recommended precautions and restrictions. From a sampling perspective, reduced concerns over having a birth support person and reducing visits in wave 2 compared to wave 1 may simply reflect that wave 2 respondents were earlier in their pregnancies (e.g., first trimester), and so they did not have the same needs or worries that those later in pregnancy might report.

Prenatal healthcare access, concerns about childbirth, and childbirth decision-making were largely the same for racially and ethnically minoritized pregnant people as compared to white, non-Hispanic pregnant people in both ‘surge’ and ‘non-surge’ contexts, after controlling for other factors. However, that broader finding is contradicted, at least descriptively, when the ‘racially minoritized’ category is disaggregated further. Black and Indigenous pregnant people were far more concerned about accessing childbirth healthcare than white, Asian/Pacific Islander, and multiracial pregnant people. The finding that individuals from historically minoritized groups reported greater childbirth concerns is not surprising. Rather, our findings reflect the established research on poorer birth outcomes in these groups [28, 29]. While maternal outcomes in general in the US rank lower than other developed countries [30], they are also worse in minoritized groups both in the US and globally [31–33]. Similarly, the pandemic has disproportionately affected people of color [34–39] and pregnant people of color [24, 40]. The long-term impacts of these combined disparities on birthing people and their offspring should be a priority for future research, including tailoring care for this population to mitigate the effects. The magnitude of the difference in childbirth concerns for our sample is quite large for both Black and Indigenous respondents, at two-thirds and half of a standard deviation, respectively, when compared to white respondents. For Black respondents, for example, this is about the same magnitude of difference as comparing those who report a great deal of difficulty paying their bills as compared to those who report no difficulty. Moreover, these groups face significantly higher economic and healthcare-related stress, which studies have shown is exacerbated in the context of the pandemic [41]. Importantly, this finding is masked when simply comparing historically minoritized groups to non-minoritized populations. Despite having more than 800 participants across the two waves, we had insufficient sample size to test for statistically significant differences across sub-groups. Future research should investigate these findings further, including studies with larger samples and those focused solely on minoritized groups.

Finally, during both the summer lull and the winter surge, more financially insecure pregnant people were more likely to face barriers to prenatal healthcare access, more likely to be concerned about childbirth healthcare access, and considered having an out-of-hospital birth more often than those with more financial security. Our study underscores previous research that shows living at or below the poverty line is associated with worse prenatal care and maternal outcomes [42, 43]. This is especially concerning given that the pandemic has decreased financial security for many Californian pregnant people [44, 45], and, despite multiple federal COVID relief bills, it is clear that these financial strains are going to persist for several years [46].

Our findings should be considered within the context of important limitations. First, as previously noted, we did not have the sample sizes necessary to disaggregate all of the findings among subgroups. This is clearly critical in future research, especially in light of ongoing disparities in perinatal outcomes among historically minoritized groups. Additionally, this survey was self-report and participants were recruited via social media. While the overall sample reflects the demographics of California (white alone 71.9%, Black or African American alone 6.5%, Asian/Pacific Islander 17.6%, Two or More Races 4.0%, Hispanic or Latino 39.4%, White alone, not Hispanic or Latino 36.5%) [47], and the survey was available in English and Spanish, there may be selection bias, and the sample cannot be considered representative of all pregnant people in the state or in the United States at large.

Despite these limitations, this study has important implications for healthcare providers and policymakers. As local, state, and federal governments continue to respond to the pandemic, it is clear that perinatal and birthing people – and subpopulations of perinatal and birthing people – should be considered a unique audience with specific needs and concerns that must be addressed. For example, when pregnant people first seek care, normalizing potential fears and then providing information about what to expect in clinic, during routine tests, and in the birth hospital that is specific to COVID prevention can help them to prepare, minimize potential concerns, and provide multiple opportunities over the course of pregnancy to ask questions. Likewise, as vaccine roll-out begins to include pregnant people, it will be critical to address the specific concerns of COVID-19 in pregnancy in order to facilitate uptake, especially among historically minoritized groups. Active efforts on the part of healthcare providers and healthcare systems to build trust in communities where they have done historical harm will be necessary to address what will likely be gaps in vaccination rates, which will only exacerbate existing disparities in COVID-19 morbidity and mortality [41, 48].

It is critical for healthcare providers and policymakers to continue focusing on addressing needs and gaps in care for historically minoritized subgroups [28, 49]. Even when overall levels of healthcare access in the two waves of our study improved over time, among Black participants, concerns over childbirth actually worsened. While the intersection of racism and perinatal morbidity and mortality certainly are on the radar for providers and policymakers, it is clear that Black and Indigenous pregnant people in our sample are experiencing more distress, possibly because rates of COVID-19 morbidity and mortality are higher in racially minoritized populations as well [41]. The combined stress of medical racism associated with birthing and COVID-19 must be addressed [20, 29]. This may include more financial investments in provider groups that primarily serve Black and Indigenous communities, as well as support for services such as community doulas and native / Indigenous approaches to birthing, which have been shown to improve perinatal outcomes in these groups [50, 51].

Lastly, as COVID relief bills attempt to address financial strain through lump sum payments, it is clear that this approach is insufficient to fill the gap that has resulted from months of poor economic conditions and recession associated with the pandemic [52]. Given that health care costs are the number one driver of financial strain, policy proposals aimed at improving healthcare coverage beyond the first year postpartum to all individuals all of the time (e.g., Medicare for All) can help to bridge the gap not only in perinatal healthcare access, but lifelong access especially among minoritized groups [53–56].

## Conclusion

Despite surging COVID-19 cases and hospitalizations during the winter of 2020–2021, pregnant Californians generally enjoyed more access to, and fewer concerns about, their perinatal healthcare compared to those surveyed COVID-19 ‘lull’ during the summer of 2020. However, across ‘surge’ and ‘non-surge’ pandemic circumstances, marginalized pregnant people continued to fare worse. This was especially true for those facing acute financial difficulty, and racially minoritized individuals identifying as Black or Indigenous. Targeting those communities for increased upstream healthcare supports – such as earlier access to vaccination, monetary investments in the healthcare structures that support these communities, and more direct financial relief to those facing the greatest economic insecurity – are promising avenues to blunt the negative impacts of the COVID-19 pandemic on pregnant people in California.

## Supplementary Information

**Additional file 1.** Participant Questionnaire.

**Additional file 2 Appendix Table 1**. Correlations between minoritization, financial strain, healthcare access, childbirth concerns, and childbirth decision-making (June–July 2020).

**Additional file 3 Appendix Table 2**. Correlations between minoritization, financial strain, healthcare access, childbirth concerns, and childbirth decision-making (December 2020–January 2021).

## Data Availability

The datasets generated and/or analyzed during the current study are not publicly available due to ongoing analyses but are available from the corresponding author on reasonable request.

## Abbreviations

SARS-CoV-2: Severe acute respiratory syndrome coronavirus 2
COVID-19: Coronavirus disease 2019
CDC: Center for Disease Control and Prevention
WHO: World Health Organization
POC: People of color
OLS: Ordinary least-squares
