# Supplementary material for: Perinatal health care access, childbirth concerns, and birthing decision-making among pregnant people in California during COVID-19
Source: BMC Pregnancy Childbirth. 2021 Jul 2;21:477. doi: 10.1186/s12884-021-03942-y (PMC8250556; doi:10.1186/s12884-021-03942-y)
Supplement: Supplementary file 1 — Additional file 1. Participant Questionnaire. [file 12884_2021_3942_MOESM1_ESM.pdf]

Impact of COVID-19 on Pregnant Women’s Preventive Health Behaviors

MAY – JULY 2020; DECEMBER 2020 – JANUARY 2021

SCREENING - 4 Questions

Q1_1 Are you currently pregnant?
1 = Yes
2 = No
3 = I prefer not to answer

Q1_2 Do you live in California?
1 = Yes
2 = No
4 = I prefer not to answer

Q1_3 Are you serving as a surrogate for another individual or couple?
1 = Yes
2 = No
3 = I prefer not to answer

Q1_4 How old are you?
Enter years: Write in

SURVEY

Q2 Do you consent to take part in this research study?
1 = I accept
2 = I do not accept

Q3_2 How would you describe the city in which you live? (SELECT ONE)
1 = Rural (e.g., town less than 2,500 people)
2 = Semi-rural (e.g., town more than 2,500 people but less than 20,000 people)
3 = Suburban (e.g., city or town more than 20,000 people but less than 250,000 people)
4 = Urban (e.g., city more than 250,000 people but less than 1,000,000 people)
5 = Major metropolitan area (e.g., city more than 1,000,000 people)
6 = I prefer not to answer

Q3_3 Are you Hispanic or Latina?
1 = Yes
2 = No
3 = I prefer not to answer

Q3_4 What is your race? (SELECT ONE)
1 = White alone
2 = Black or African American
3 = American Indian or Alaska Native alone
4 = Asian alone
5 = Native Hawaiian or Other Pacific Islander alone
6 = Some other race alone
7 = Two or more races
8 = I prefer not to answer

Q3_9 Is your partner an essential employee?
1 = Yes
2 = No
3 = I prefer not to answer

Q3_13 How would you describe your employment status? (Select one)
1 = Work full-time
2 = Work part-time
3 = Not currently employed but seeking work
4 = Not currently employed but not seeking work
5 = Student
6 = Other: Write in
7 = I prefer not to answer

Q3_13TEXT How would you describe your employment status? (Select one)
Other: Write In - Text

BRANCH LOGIC TO WORKING FULL-TIME:
Q3_14 (BRANCH) Are you an essential employee?
1 = Yes
2 = No
3 = I prefer not to answer
0 = Does not work full time

Q3_22 During the past 2 months, how much difficulty have you had paying your bills?
1 = A great deal of difficulty
2 = Quite a bit of difficulty
3 = Some difficulty
4 = A little difficulty
5 = No difficulty at all
6 = I prefer not to answer

Pregnancy Info

Q4_1w How far along are you?
Enter weeks

Q4_1d How far along are you?
Enter days

Q4_2 Are you pregnant with a single baby or multiple babies?
1 = Single
2 = Multiple
4 = I don't know
0 = didn't answer

Q4_3 Have you seen your provider for this pregnancy?
1 = Yes
2 = No

3 = I prefer not to answer

Q4_5 (BRANCH) How many prenatal visits have you had so far?
Enter #

Q4_7 (BRANCH) Has your provider started doing remote visits, such as using video or telephone?
1 = Yes
2 = No
3 = I don't know
4 = I prefer not to answer

Q4_8 (BRANCH) Has your provider reduced the number of visits?
1 = Yes
2 = No
3 = I don't know
4 = I prefer not to answer

Q4_11 Where do you plan to delivery your baby?
1 = Hospital
2 = Birthing center
3 = Home
4 = Other
5 = I prefer not to answer

Q4_11TEXT Other – text

Q4_12 Did COVID-19 change your plans for where to deliver you baby?
1 = Yes
2 = No
3 = I prefer not to answer

Q4_16 Is this your first pregnancy?
1 = Yes
2 = No
3 = I prefer not to answer

BRANCH LOGIC FOR NO:
Q4_17 (BRANCH) How many times have you been pregnant?
Write in #

Q4_18 (BRANCH) How many biological children do you have?
Write in #

Q4_19 (BRANCH) How many miscarriages have you had?
Write in #

Q4_20 How many stillbirths have you had?
Write in #

Questions About the Covid19 Pandemic

We are interested in knowing how you manage your daily routine, health habits, and your general well-being during this global pandemic. Please rate the following statements in terms of how you have been doing for the last 7 days.

1 = Does not apply/I don't know
2 = Never (0 days)
3 = Rarely (1-2 days)
4 = Sometimes (3-4 days)
5 = Often (5-6 days)
6 = Always (7 days)
7 = I prefer not to answer

Q10_1 I take care of my mental health and well-being as I always have.
Q10_2 I take care of my physical health and well-being as I always have.
Q10_3 I stay inside my apartment, house or place where I live for most of the day.
Q10_4 I seek care for health problems not related to my pregnancy.
Q10_5 I schedule an extra visit with my prenatal provider if I am concerned about my pregnancy.
Q10_6 I do kick counts.
Q10_7 I make sure to get outside for at least 30 minutes.
Q10_8 I get at least 30 minutes of moderate activity (e.g., brisk walk, easy jog, general gardening).
Q10_9 I get at least 30 minutes of vigorous activity (e.g., uphill or race walk, running).
Q10_10 I avoid the news.
Q10_11 I have someone else do my shopping for me.
Q10_12 I have someone else do my errands for me.
Q10_13 I eat sweets (e.g., cookies, candy).
Q10_14 I eat salty snacks (e.g., chips, crackers).
Q10_15 I eat when I'm hungry.
Q10_16 I pay attention to healthy nutrition when I make food choices.
Q10_17 I worry that we will run out of food and will not have enough money to pay for more.
Q10_18 I drink alcohol.
Q10_19 I use marijuana.
Q10_20 I use CBD products.
Q10_21 I smoke cigarettes.
Q10_22 I use other tobacco products (e.g., e-cigarettes, snuff, chew).
Q10_23 I drink caffeinated beverages.
Q10_24 I drink sugary drinks (e.g., soda, flavored juices.
Q10_25 I am living my life as I did before the COVID pandemic.
Q10_26 My sleep has been interrupted.
Q10_27 I am sleeping less than usual.
Q10_28 I am sleeping more than usual.
Q10_29 I wake up frequently (more than 2 times) in the middle of the night.

Q10_30 It takes me 15 minutes or more to fall asleep at night.
Q10_31 I sleep at least 7 hours every night.
Q10_32 I feel rested when I wake up.
Q10_33 I worry how to shelter-in-place with a newborn.
Q10_34 I wear a mask when I go out in public.
Q10_35 Family members who live with me wear masks when they go out in public.
Q10_36 I let family and friends visit.
Q10_37 I make sure to stay at least 6 feet away from people if I am out in public.
Q10_38 I order all groceries online.
Q10_39 I order all non-food items online.
Q10_40 I cook at home for most meals.
Q10_41 I worry about my family financial situation.
Q10_42 I take my prenatal vitamin daily.
Q10_43 I wipe down frequently used areas in my house or apartment with Clorox wipes or other sanitizing solutions.
Q10_44 I wipe down groceries and other items I purchase before using them.
Q10_45 I wipe down groceries and other items I have shipped or delivered before using them.
Q10_46 I wash my hands before eating.
Q10_47 I wash my hands when I come in from outside.
Q10_48 I have the support I need from family.
Q10_49 I have the support I need from my friends.
Q10_50 I spend more than 2 hours a day on social media (e.g., Twitter, Facebook, Instagram)
Q10_51 I stock up on essential items.
Q10_52 I understand the actions I should take to reduce my risk of getting sick from the coronavirus/COVID19
Q10_53 I worry about getting sick.
Q10_54 I worry that I will be admitted to the hospital.
Q10_55 I worry about losing my pregnancy.
Q10_56 I worry that I'm going to die.
Q10_57 I worry that my baby will be born early.
Q10_58 I worry about my baby getting sick.
Q10_59 I worry that my baby will have to stay in the hospital after she or he is born.
Q10_60 I worry that my baby will die.
Q10_61 I worry that I will not have my birth support person with me in the delivery room.
Q10_62 I worry that my provider or health care team will not be available during my delivery.
Q10_63 I worry that my healthcare team will not have the equipment and resources they need to support my delivery.
Q10_64 I am thinking about not having my baby in a hospital.
