# Supplementary material for: Perinatal health care access, childbirth concerns, and birthing decision-making among pregnant people in California during COVID-19
Source: BMC Pregnancy Childbirth. 2021 Jul 2;21:477. doi: 10.1186/s12884-021-03942-y (PMC8250556; doi:10.1186/s12884-021-03942-y)
Supplement: Supplementary file 2 — Additional file 2 Appendix Table 1. Correlations between minoritization, financial strain, healthcare access, childbirth concerns, and childbirth decision-making (June–July 2020). [file 12884_2021_3942_MOESM2_ESM.pdf]

Appendix Table 1: Correlations between minoritization, financial strain, healthcare access, childbirth concerns, and childbirth decision-making (June-July 2020)

	1	2	3	4	5	6	7	8	9	10	11	12
1. White (non-minoritized)	1.00											
2. Hispanic	-0.42 ***	1.00										
3. Financially insecure	-0.14 **	0.26 ***	1.00									
4. Concern: birth support person	-0.07	0.07	0.16 **	1.00								
5. Concern: provider unavailable	-0.12 *	0.11 *	0.19 ***	0.38 ***	1.00							
6. Concern: lack of resources during birth	-0.14 **	0.04	0.25 ***	0.37 ***	0.56 ***	1.00						
7. Remote visits started	-0.04	-0.01	0.02	-0.01	-0.08	-0.03	1.00					
8. Prenatal visits reduced	-0.05	0.04	0.18 ***	0.06	0.12 *	0.15 **	0.37 ***	1.00				
9. Seek healthcare outside pregnancy	0.01	0.07	0.06	0.06	0.10 [t]	0.10 *	-0.05	0.03	1.00			
10. Scheduling extra visits if concerned	-0.04	-0.04	0.10 *	-0.03	0.14 **	0.11 *	0.02	-0.01	0.29 ***	1.00		
11. Considering out-of-hospital birth	-0.06	0.03	0.19 ***	0.05	0.15 **	0.26 ***	0.00	0.18 ***	0.14 **	0.08	1.00	
12. Planning out-of-hospital birth	0.07	-0.08	-0.07	-0.18 ***	-0.12 *	-0.04	-0.01	0.03	0.03	0.02	0.36 ***	1.00

Note: [t]p<0.10, *p<0.05, **p<0.01, ***p<0.001
