# Supplementary material for: Perinatal health care access, childbirth concerns, and birthing decision-making among pregnant people in California during COVID-19
Source: BMC Pregnancy Childbirth. 2021 Jul 2;21:477. doi: 10.1186/s12884-021-03942-y (PMC8250556; doi:10.1186/s12884-021-03942-y)
Supplement: Supplementary file 3 — Additional file 3 Appendix Table 2. Correlations between minoritization, financial strain, healthcare access, childbirth concerns, and childbirth decision-making (December 2020–January 2021). [file 12884_2021_3942_MOESM3_ESM.pdf]

Appendix Table 2: Correlations between minoritization, financial strain, healthcare access, childbirth concerns, and childbirth decision-making (December 2020-January 2021)

	1	2	3	4	5	6	7	8	9	10	11	12
1. White (non-minoritized)	1.00											
2. Hispanic	-0.23***	1.00										
3. Financially insecure	-0.18***	0.26***	1.00									
4. Concern: birth support person	-0.07	0.09	0.29***	1.00								
5. Concern: provider unavailable	-0.07	0.04	0.22***	0.52***	1.00							
6. Concern: lack of resources during birth	-0.02	-0.05	0.22***	0.46***	0.64***	1.00						
7. Remote visits started	-0.01	0.08	-0.02	-0.06	-0.08	-0.04	1.00					
8. Prenatal visits reduced	-0.08	0.04	0.08	0.15*	0.10	0.09	0.40***	1.00				
9. Seek healthcare outside pregnancy	0.07	-0.04	-0.08	-0.13*	-0.03	-0.07	0.11	-0.01	1.00			
10. Scheduling extra visits if concerned	-0.01	-0.05	0.07	-0.05	0.08	0.03	0.07	-0.02	0.27***	1.00		
11. Considering out-of-hospital birth	0.01	-0.11	0.07	0.12*	0.15**	0.11*	0.00	0.09	0.02	0.02	1.00	
12. Planning out-of-hospital birth	-0.02	-0.11	0.03	-0.07	-0.06	-0.04	0.03	-0.02	-0.03	0.06	0.33***	1.00

Note: $^{t}p<0.10$, *p<0.05, **p<0.01, ***p<0.001
